# GC-MS Analysis and Inhibitory Evaluation of *Terminalia catappa* Leaf Extracts on Major Enzymes Linked to Diabetes

**DOI:** 10.1155/2019/6316231

**Published:** 2019-09-05

**Authors:** Franklyn Nonso Iheagwam, Emmanuel Nsedu Israel, Kazeem Oyindamola Kayode, Opeyemi Christianah De Campos, Olubanke Olujoke Ogunlana, Shalom Nwodo Chinedu

**Affiliations:** ^1^Department of Biochemistry, Covenant University, Canaanland, P.M.B. 1023 Ota, Ogun State, Nigeria; ^2^Covenant University Public Health and Wellbeing Research Cluster (CUPHWERC), Covenant University, Canaanland, P.M.B. 1023 Ota, Ogun State, Nigeria

## Abstract

*Terminalia catappa* leaves are used in managing both diabetes mellitus and its complications in Southwest Nigeria. However, its inhibitory activity on enzymes implicated in diabetes is not very clear. This study investigated the *in vitro* inhibitory properties and mode of inhibition of *T. catappa* leaf extracts on enzymes associated with diabetes. The study also identified some bioactive compounds as well as their molecular interaction in the binding pocket of these enzymes. Standard enzyme inhibition and kinetics assays were performed to determine the inhibitory effects of aqueous extract (TCA) and ethanol extract (TCE) of *T. catappa* leaves on *α*-glucosidase and *α*-amylase activities. The phytoconstituents of TCA and TCE were determined using GC-MS. Molecular docking of the phytocompounds was performed using Autodock Vina. TCA and TCE were the most potent inhibitors of *α*-glucosidase (IC_50_ = 3.28 ± 0.47 mg/mL) and *α*-amylase (IC_50_ = 0.24 ± 0.08 mg/mL), respectively. Both extracts displayed a mixed mode of inhibition on *α*-amylase activity, while mixed and noncompetitive modes of inhibition were demonstrated by TCA and TCE, respectively, on *α*-glucosidase activity. The GC-MS analytic chromatogram revealed the presence of 24 and 22 compounds in TCE and TCA, respectively, which were identified mainly as phenolic compounds, terpenes/terpenoids, fatty acids, and other phytochemicals. The selected compounds exhibited favourable interactions with the enzymes compared with acarbose. Overall, the inhibitory effect of *T. catappa* on *α*-amylase and *α*-glucosidase may be ascribed to the synergistic action of its rich phenolic and terpene composition giving credence to the hypoglycaemic nature of *T. catappa* leaves.

## 1. Introduction

Diabetes mellitus (DM) is an endocrine, chronic, noncommunicable disease plaguing the world populace with a rapid increase. A reported 425 million individuals were globally affected by DM, while 629 million people have been projected to be affected by 2045 [1]. DM is characterized by hyperglycaemia as a consequence of impaired insulin secretion (as experienced in type 1 diabetes) or insulin resistance (as experienced in type 2 diabetes) resulting in diabetic complications such as diabetic retinopathy, neuropathy, and nephropathy [2]. Type 2 diabetes (T2D) is the most prevalent type of DM affecting over 90% of people diagnosed with this disease [3]. Lifestyle modification through exercise and diet as well as oral medications such as metformin, pioglitazone, and acarbose to decrease hepatic glucose output and insulin sensitivity improvement and reduce starch digestibility, respectively, are management methods currently employed in T2D [4].

*Terminalia catappa* Linn, commonly known as Indian almond, belongs to the Combretaceae family and grows in the tropics of Asia, Africa, and Australia [5]. In urban regions where these trees are found, the leaves form a menace and are the major constituents of generated lignocellulosic waste. In Southwest Nigeria, it is commonly called “igi furutu” or “igifuruntu,” and various plant parts are used to treat diabetic complications by the locals [6]. Several studies have reported different activities of *T. catappa* extracts such as hepatoprotective effects, anticancer property, antimutagenic activity, and antiaging property [7]. Divya and Anand [8] have also reported on the inhibitory property of *T. catappa* methanolic leaf extract on diabetic-linked enzymes. Despite this antidiabetic claim by the locals, the elaborate antidiabetic mechanism is far from clear. This study assessed the inhibitory properties of *T. catappa* leaf extracts on *α*-glucosidase and *α*-amylase, the mode of enzyme inhibition, as well as identified phytocompounds present and proposed the molecular mechanism of binding in the active sites of the enzymes.

## 2. Materials and Methods

### 2.1. Materials

*α*-Glucosidase, *α*-amylase enzymes, and their substrates were acquired from Solarbio Life Sciences, Beijing, China. Other chemicals were products of Sigma-Aldrich, St. Louis, USA.

### 2.2. Plant Collection, Identification, and Extraction

Mature *T. catappa* leaves were sourced between October and December 2016, from Covenant University compound. They were identified by Dr. J. O. Popoola of Biological Sciences Department and voucher specimen deposited at Biological Sciences Department herbarium, Covenant University, Ota, Ogun State, with herbarium number TC/CUBio/H809. Aqueous *T. catappa* (TCA) and ethanol *T. catappa* (TCE) leaf extracts were prepared as reported by Iheagwam et al. [9]. The leaves were cut, air-dried, pulverised, and macerated in distilled water and ethanol (80%), respectively, at 1 : 10 (w/v) ratio for 72 hrs. The obtained filtrates were concentrated using a rotary evaporator.

### 2.3. Antidiabetic Assessment

#### 2.3.1. *α*-Glucosidase Inhibitory Activity

*α*-Glucosidase inhibitory activity of the extracts was evaluated according to the method described by Ibrahim and Islam [10] with slight modification. Various extract concentration and acarbose (1–5 mg/mL, 250 *μ*L) were incubated at 37°C for 15 min with *α*-glucosidase solution (1 U/mL, 500 *μ*L). *ρ*-Nitrophenyl-*α*-D-glucopyranoside (pNPG) solution (5 mM, 250 *μ*L) was thereafter added, and the resulting mixture was incubated for 20 min at 37°C. The reaction was terminated by adding Na_2_CO_3_ (0.2 M, 100 *μ*L), and absorbance was measured at 405 nm. Phosphate buffer (100 mM) was used as control in place of inhibitors. Inhibitory activity was calculated using the following equation:

(1) $$\begin{array}{c} \%\text{ inhibition }=100\times \left(\frac{A_{\text{c}}-A_{\text{s}}}{A_{\text{c}}}\right), \end{array}$$

where *A*_s_ = absorbance in the presence of sample and *A*_c_ = absorbance of control. All solutions were prepared in 0.1 M phosphate buffer (pH 6.8).

The method of Sabiu and Ashafa [11] was adopted for *α*-glucosidase inhibitory kinetics. Extract (5 mg/mL, 250 *μ*L) was preincubated with *α*-glucosidase solution (1 U/mL, 500 *μ*L) for 10 min at 25°C. Varying pNPG concentrations (0.15–5 mg/mL, 250 *μ*L) were added and incubated for 10 min at 25°C to both sets of reaction mixtures to start the reaction. Thereafter, Na_2_CO_3_ (0.2 M, 500 *μ*L) was added to stop the reaction. For the control kinetic reaction, 100 mM phosphate buffer (pH 6.8, 250 *μ*L) was used in place of the extract. Reaction rates (*v*) were calculated, and double reciprocal plots of *α*-glucosidase inhibition kinetics were determined.

#### 2.3.2. *α*-Amylase Inhibitory Activity

*α*-Amylase inhibitory activity of the extracts was evaluated by adopting the method described by Ibrahim and Islam [10] with slight modification. Various extract concentrations (1–5 mg/mL, 250 *μ*L) and acarbose were incubated at 37°C for 20 min with amylase solution (2 U/mL, 500 *μ*L). Starch solution (1%, 250 *μ*L) was later added to the reaction mixture and incubated at 37°C for 1 h. Dinitrosalicylic acid (DNS) colour reagent (1 mL) was added to stop the reaction. The resulting mixture was boiled for 10 min, and absorbance was measured at 540 nm. Phosphate buffer (100 mM) was used as control in place of inhibitors. The *α*-amylase inhibitory activity was calculated using the following formula:

(2) $$\begin{array}{c} \text{\% inhibition }=100\;\times \left(\frac{A_{\text{c}}-A_{\text{s}}}{A_{\text{c}}}\right), \end{array}$$

where *A*_s_ = absorbance in the presence of sample and *A*_c_ = absorbance of control. All solutions were prepared in 100 mM phosphate buffer (pH 6.8).

The method of Sabiu and Ashafa [11] was adopted for *α*-amylase inhibitory kinetics. In brief, extract (250 *μ*L, 5 mg/mL) was incubated with *α*-amylase (2 U/mL, 500 *μ*L) for 10 min, before the addition of various substrate concentrations (0.3–10 mg/mL, 250 *μ*L). The reaction proceeded as highlighted for *α*-glucosidase. *α*-Amylase inhibition kinetics was determined from the Lineweaver–Burk double reciprocal plot.

### 2.4. Gas Chromatography-Mass Spectroscopy (GC-MS) Analysis

The GC-MS analysis of *T. catappa* extracts was carried out using GCMS-QP2010SE SHIMADZU, Japan, fused with the Optima 5 ms capillary column (30 × 0.25 mm) of 0.25 *μ*m film thickness following the described method of Ajiboye et al. [12] with slight modifications. The gas chromatography conditions were as follows: pure helium carrier gas (flow rate: 1.56 mL/min; linear velocity: 37 cm/s), initial column oven temperature (60°C) programmed to increase to 160°C at the rate of 10°C/min and then finally to 250°C with a hold time of 2 min/increment, and an injection volume of 0.5 *μ*L in the splitless mode with a split ratio of 1 : 1 and injector temperature set at 200°C. Mass spectrophotometer conditions were as follows: ion source temperature (230°C), interface temperature (250°C), solvent delay at 4.5 min, and acquisition in a scan range of 50–700 amu. Electron ionization mode and multiplier voltage were set at 70 eV and 1859 V, respectively. Retention time, fragmentation pattern, and mass spectral data of the unknown components in the extracts were compared with those in Wiley and National Institute of Standards and Technology (NIST) libraries for compound identification.

### 2.5. In Silico *α*-Glucosidase and *α*-Amylase Inhibition Prediction

#### 2.5.1. Ligand and Protein Modelling

The structures of the GC-MS identified compounds with ≥5% abundance were prepared as reported by Iheagwam et al. [13]. The 3D structure of *α*-glucosidase and *α*-amylase was modelled using the crystal structures with PDB codes 5kzw and 1b2y, respectively, obtained from RCSB protein data bank as templates in SWISS-MODEL [14].

#### 2.5.2. Virtual Screening, Drug-Likeness, and Molecular Docking

Virtual screening of selected identified ligands, analysis of drug-likeness using the rule of five (RO5), and molecular docking were carried out according to the methodology of Iheagwam et al. [13]. However, grid dimensions of the binding pockets were 60 × 40 × 32 and 40 × 34 × 40 points separated by 1 Å for *α*-glucosidase and *α*-amylase, respectively. Inhibition constant (*K*_*i*_) of docked ligands were calculated by using the following formula:

(3) $$\begin{array}{c} K_{i}=10^{\left[\text{binding energy }\left(\text{BE}\right)/1.366\right]}. \end{array}$$

### 2.6. Statistical Analysis

Data were analysed using SPSS version 25 (IBM Corp., New York, USA) and subjected to one-way analysis of variance (ANOVA) using the Duncan multiple range post hoc test. Values were reported as mean ± standard deviation (SD) of three (3) replicates and considered significantly different at *p* < 0.05.

## 3. Results

**Figure 1:**
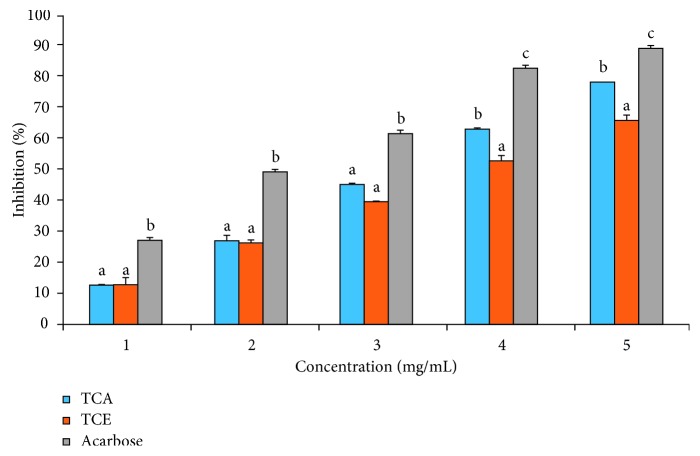
*T. catappa* leaf extract inhibitory effect on *α*-glucosidase activity. Bars are expressed as means ± SD of triplicate determinations. Bars with different superscripts on each concentration denote significant difference (*p* < 0.05).

**Figure 2:**
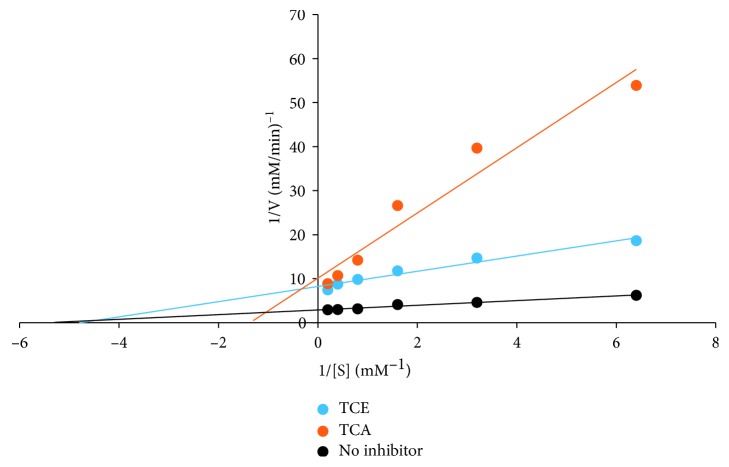
*T. catappa* leaf extract mode of inhibition on *α*-glucosidase activity.

For the *α*-glucosidase inhibitory activity of TCA and TCE as shown in Figure 1, a significantly (*p* < 0.05) lower inhibition by the extracts was observed at all concentrations relative to control. TCA exhibited a significantly (*p* < 0.05) higher inhibition of *α*-glucosidase activity compared to TCE. Nonetheless, at lower concentrations (1–3 mg/mL), there was no difference between the inhibitory activities of TCA and TCE. These were further supported by a lower IC_50_ value (2.23 ± 0.21 mg/mL) for acarbose when compared with TCA (3.28 ± 0.47 mg/mL) and TCE (3.78 ± 0.26 mg/mL (Table 1). The kinetic study on the inhibition mode using the double reciprocal plot revealed TCE exhibited a noncompetitive mode of inhibition with a common *K*_m_ value of 0.19 mM and *V*_max_ value of 0.13 mM/min, while TCA exhibited a mixed mode of inhibition with a *K*_m_ value of 0.77 mM and *V*_max_ value of 0.1 mM/min (Figure 2).

**Figure 3:**
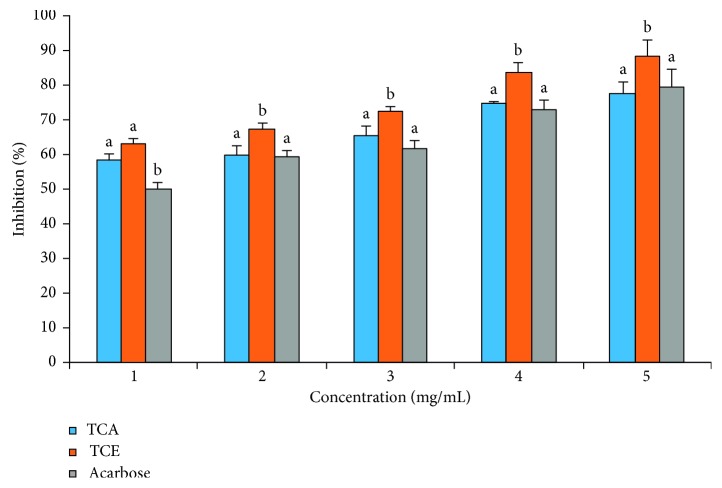
*T. catappa* leaf extract inhibitory effect on *α*-amylase activity. Bars are expressed as means ± SD of triplicate determinations. Bars with different superscripts on each concentration denote significant difference (*p* < 0.05).

**Figure 4:**
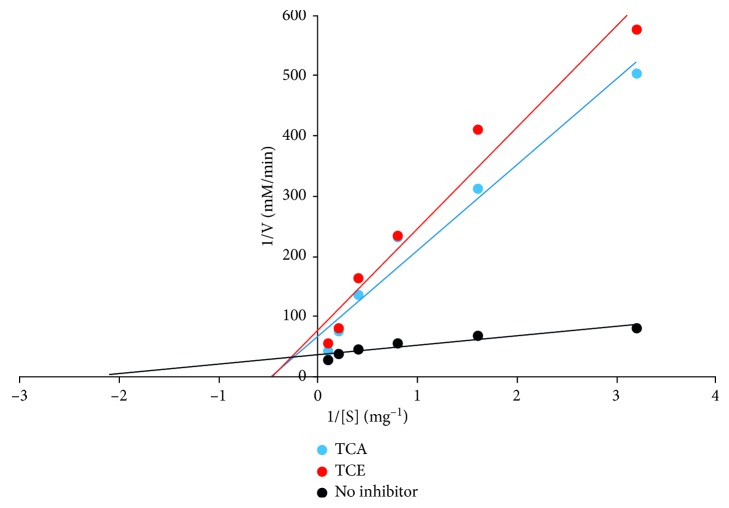
*T. catappa* leaf extract mode of inhibition on *α*-amylase activity.

The percentage inhibition of *α*-amylase activity by *T. catappa* leaf extracts is presented in Figure 3. Though a concentration-dependent effect was observed, TCE inhibitory activity was significantly (*p* < 0.05) higher than TCA and acarbose at all concentrations. TCA elicited inhibitory effects that competed favourably with the standard drug (acarbose). These results were supported with an IC_50_ of 0.24 ± 0.08, 0.75 ± 0.14, and 0.85 ± 0.18 mg/mL recorded for TCE, TCA, and acarbose, respectively (Table 1). TC extracts displayed a mixed mode of inhibition on *α*-amylase activity with a *V*_max_ value of 0.013 and 0.016 mM/min and *K*_m_ values of 2.27 and 2.22 mg for TCE and TCA, respectively (Figure 4), from the Lineweaver–Burk double reciprocal plot.

**Figure 5:**
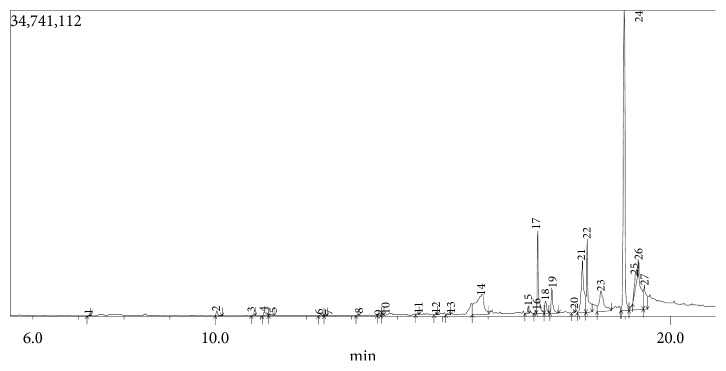
GC chromatogram of *T. catappa* ethanolic leaf extract.

**Figure 6:**
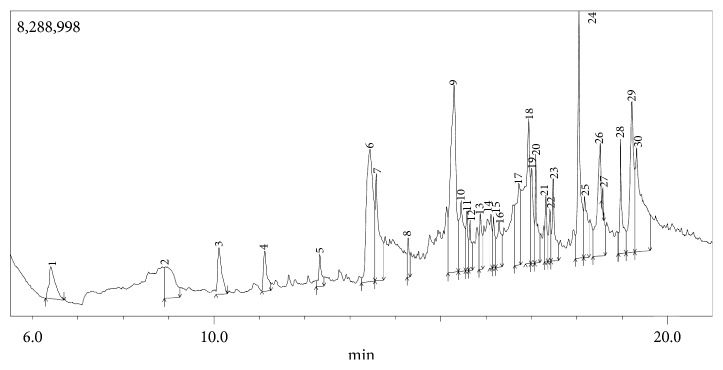
GC chromatogram of *T. catappa* aqueous leaf extract.

The GC-MS chromatogram as shown in Figures 5 and 6 confirmed the presence of various phytochemicals with different retention times for TCE and TCA, respectively. A total of 27 and 29 peaks were identified in TCE and TCA chromatograms, respectively.

The identified phytochemicals present in TCE and TCA are shown in Tables 2 and 3, respectively, based on their retention time, abundance, and compound classification. GC-MS analysis revealed the presence of 24 compounds in TCE and 22 compounds in TCA. Seven compounds were found in both extracts; however, phytol and *n*-hexadecanoic acid were higher in TCE, while 4H-pyran-4-one, 2,3-dihydro-3,5-dihydroxy-6-methyl-, benzofuran, 2,3-dihydro-, 2-methoxy-4-vinylphenol, and 9,12-octadecadienoic acid (Z,Z)- were higher in TCE. It was also observed that there was no much difference in the abundance of vitamin E in both extracts.

For TCE, 9, 26, 13, 30, and 25% of the identified compounds were classified as carbohydrates, fatty acids, hydrocarbons, phenolics, and terpenes/terpenoids, respectively (Table 2), while for TCA, 5, 5, 33, 33, 19, and 5% of the identified compounds were classified as alcohols, alkaloids, fatty acids, phenolics, terpenes/terpenoids, and pyrethrin, respectively (Table 3).

From the GC-MS analyses as shown in Tables 2 and 3, 12 identified compounds were found to have an abundance of 5% or more. They ranged from [1,1′-bicyclopropyl]-2-octanoic acid, 2′-hexyl-, methyl ester (5.2%), to phytol (29.54%). Virtual screening results revealed these compounds had relatively lower binding energy than acarbose (−126.81) when docked in the binding site of *α*-amylase. However, only vitamin E (−82.91) and ethyl-*α*-D-glucopyranoside (−78.11) were relatively comparable with the standard (Table 4).

As illustrated in Table 5, the same observation was also made after screening the compounds in the binding site of *α*-glucosidase. Besides ethyl-*α*-D-glucopyranoside (−79.92) and vitamin E (−89.64), *n*-hexadecanoic acid (−81.89) and phytol (−80.87) binding affinities were also comparable with acarbose (−115.55).

When the hit compounds were screened for their drug-likeness, they all obeyed Lipinski's RO5. However, phytol and vitamin E, on the one hand, violated only the octanol-water partition coefficient due to higher values than the RO5 threshold as presented in Table 6. Acarbose, on the other hand, violated 3 variants.

The binding affinity of the selected compounds as shown in Table 7 using Autodock Vina ranged from −6.0 to 8.0 kcal/mol and −5.1 to 5.9 kcal/mol for *α*-amylase and *α*-glucosidase, respectively. These values though lower were comparable with acarbose where −8.3 was recorded for *α*-amylase and −7.4 for *α*-glucosidase. Concomitantly, 1.39 to 40.51 *μ*M was the *α*-amylase inhibition constant (*K*_*i*_) recorded for the compounds compared to 0.84 *μ*M for acarbose, while 47.95 to 184.70 *μ*M was the *α*-glucosidase *K*_*i*_ recorded for the compounds compared to 3.83 *μ*M for acarbose.

**Figure 7:**
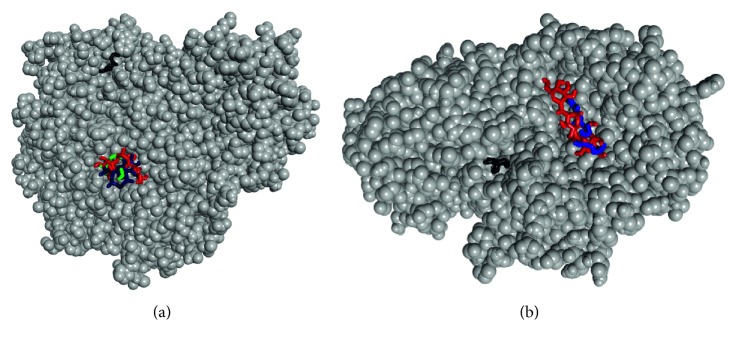
Binding of ligands in the active and allosteric pockets of (a) *α*-glucosidase and (b) *α*-amylase. The ligands ethyl-*α*-D-glucopyranoside, vitamin E, *n*-hexadecanoic acid, phytol, and acarbose were colour coded as black, blue, purple, green, and red, respectively.

**Figure 8:**
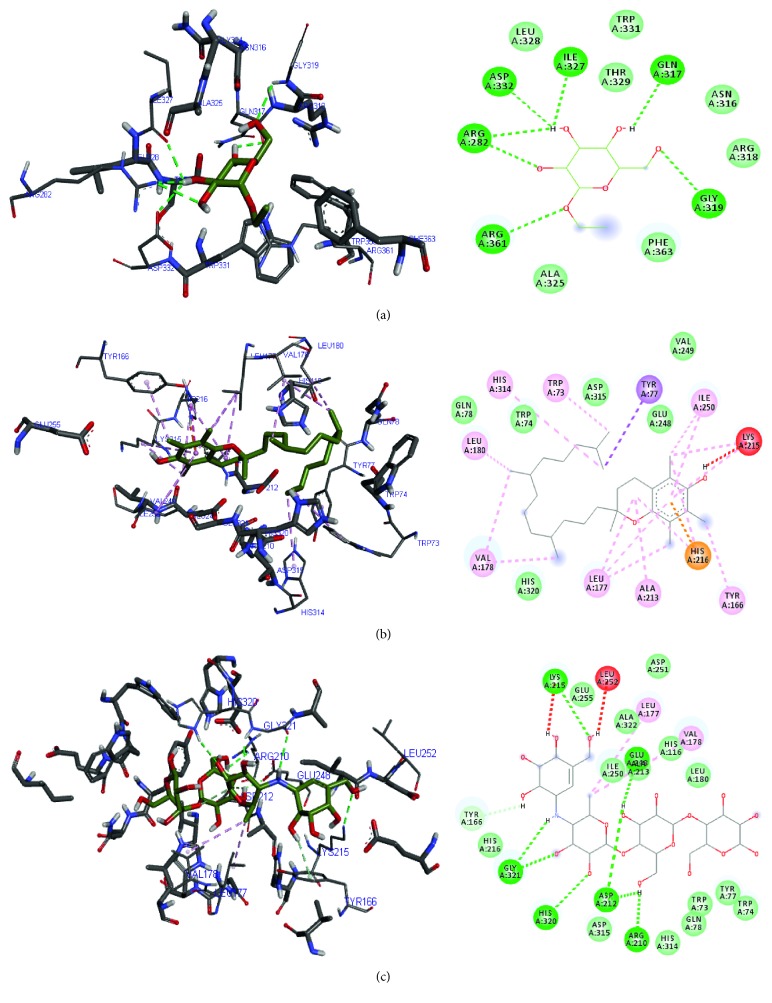
3D and 2D diagram of (a) ethyl-*α*-D-glucopyranoside, (b) vitamin E, and (c) acarbose in their *α*-amylase binding pocket using Autodock Vina. Green and blue broken lines represent conventional and carbon-hydrogen bonds, respectively; magenta, purple, and orange represent *π* bonds, while red broken lines represent unfavourable bonds.

**Figure 9:**
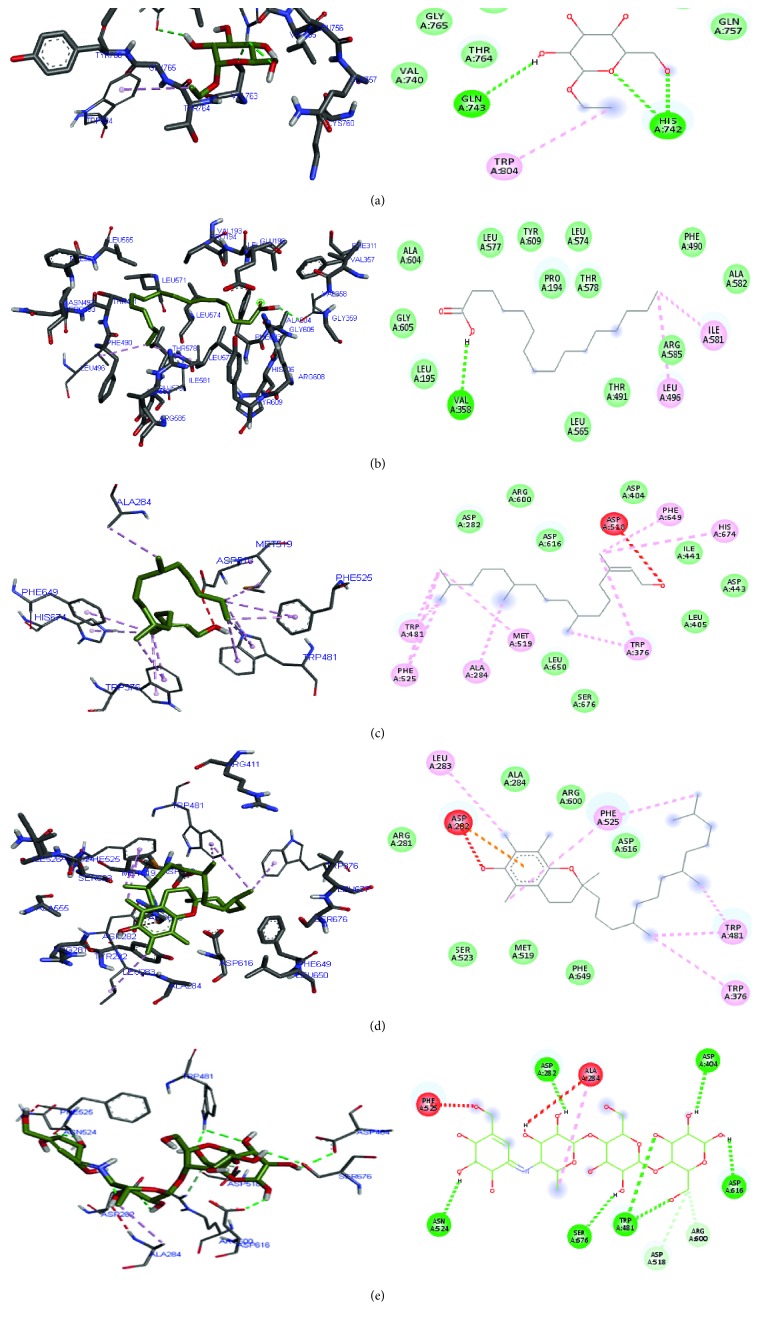
3D and 2D diagram of (a) ethyl-*α*-D-glucopyranoside, (b) *n*-hexadecanoic acid, (c) phytol, (d) vitamin E, and (e) acarbose in their *α*-glucosidase binding pocket using Autodock Vina. Green and blue broken lines represent conventional and carbon-hydrogen bonds, respectively, while magenta and red broken lines represent *p* and unfavourable bonds.

As depicted in Figure 7, the ligands bound to both the active and allosteric sites of the enzymes. It further justified the *in vitro* results as the majority of the ligands favoured active site binding compared to the allosteric site. Hydrogen, van der Waals, and *π* bonds were the common interactions displayed between the compounds and amino acids present in the binding sites of the enzymes. Trp 73, Trp 74, Tyr 77, Tyr 166, and Ile 250 were common amino acids stabilising the binding of vitamin E and acarbose in the binding pocket of *α*-amylase, while in the *α*-glucosidase binding pocket, Ala 284, Asp 616, and Trp 481 were common amino acids stabilising phytol, vitamin E, and acarbose (Figures 8 and 9).

## 4. Discussion

*α*-Glucosidase and *α*-amylase are major enzymes that metabolise carbohydrate in the digestive tract thereby affecting carbohydrate metabolism. Drugs which illicit their pharmacological action by inhibiting these enzymes are used as therapeutic control in managing diabetes through the control of postprandial hyperglycaemia [15, 16]. Research on inhibitors of these enzymes especially from medicinal plants has been intensified due to their claim of being inexpensive and less toxic compared to synthetically derived medications such as acarbose and miglitol with similar mechanisms of action [17]. Promising inhibitory activity of *T. catappa* leaf extracts was exhibited on *α*-glucosidase and *α*-amylase as previously reported in a dose-dependent manner [8]. Nonetheless, this potential was more portrayed in *α*-amylase activity as *T. catappa* leaf extracts exhibited a better inhibitory potential than acarbose. This was corroborated by various studies that have previously reported a higher inhibitory potential of medicinal plant extracts than acarbose [16, 18, 19]. It was also noteworthy that our extracts had better *α*-glucosidase and *α*-amylase inhibitory activities than those reported for *Nicotiana tabacum* and *Calotropis procera* leaf extracts [20, 21]. The reported *α*-glucosidase and *α*-amylase inhibitory activities of *Sutherlandia montana* and *Aerva lanata* (ethanol) leaf extracts were higher than our extracts except for *A. lanata* aqueous leaf extract *α*-amylase inhibitory activity which was reported to be lower than ours [4, 22]. Contrary to the reports of Xu et al. [23] and Wan et al. [24], the inhibitory activity of *T. catappa* leaf extracts was higher on *α*-amylase than on *α*-glucosidase at the varied concentrations and may be attributed to the different mechanism of action on these enzymes. This was further buttressed by the kinetic studies, where the TC extracts exhibited a mixed mode of inhibition on *α*-amylase, while mixed and uncompetitive inhibition mechanisms were observed for TCA and TCE, respectively, on *α*-glucosidase. The mixed mechanisms exhibited by TCA and TCE may suggest the bioactives present in the extracts may bind in the active site of these enzymes thereby reducing the affinity of the substrate [25, 26]. Binding of these phytochemicals in the allosteric site is also a possible mechanism of action which may lead to a conformational change of these enzymes leading to a reduction in substrate affinity for the active site concomitantly hampering enzyme catalysis [25, 26]. The results suggest these extracts may have more affinity for the enzyme (E) than the enzyme-substrate complex (ES). The noncompetitive inhibition by TCE would suggest the phytochemicals present in the extract are noncompetitive and thus would bind to a site different from the *α*-glucosidase active site affecting catalysis without having an effect on substrate binding in the active site [27]. The observed inhibitory action observed for TC extracts may be attributed to the synergistic action of identified phytochemicals from the gas chromatogram. Fatty acids, phenolic compounds, and terpenes/terpenoids were the majority classes of identified phytochemicals in both extracts. Phenolic compounds and terpenoids have also been reported to elicit antioxidant properties and alleviate oxidative stress accumulation, in the process preventing the progression of diabetic complications [28]. Compounds such as phytol [29, 30], various terpenes and terpenoids [11], hexadecanoic acid, ethyl ester, and 9,12-octadecadienoic acid (Z,Z)- [31] have been reported to exhibit various antidiabetic activities. Furthermore, reports have it that hydrolysis of phenolic compounds leads to the generation of shorter phenolic groups which accumulate, reduce oxidative stress, and inhibit amylase activity as well as other digestive enzymes reducing starch digestion [28, 32, 33]. This could also explain the better amylase inhibitory property of the extracts when compared with the glucosidase inhibitory activity. Pharmaceutical industries use structure-based drug design to solve challenges affecting integrated and classical drug design [34]. In lead compound development, compliance of test compound physicochemical properties (molecular mass, number of hydrogen bond donors and acceptors and so on) to Lipinski rule of 5 (RO5) is imperative to avoid failure during clinical trials [35, 36]. Compounds that pass RO5 (usually with none or one default) are predicted to have optimal pharmacokinetic properties, consequently subjecting them further to molecular docking [13]. Since all compounds passed RO5, they may exhibit good pharmacokinetic properties. Molecular docking further gave us a better understanding of the binding interaction between some identified phytochemicals and the key carbohydrate hydrolysing enzymes. The relatively lower binding affinity and inhibitory constant of the individual bioactives than acarbose could be due to the lesser number of hydrogen bonds present between the amino acids and the hydrogen donor/acceptor atoms in the ligands. This finding was contrary to what Pérez-Nájera et al. [37] reported on *Smilax aristolochiifolia* root extract and its compounds where the number of hydrogen bonds did not affect binding affinity. Vitamin E had the lowest free energy and *K*_*i*_ in amylase and glucosidase binding pockets which was comparable to acarbose. Consequently, it exhibited a more stable affinity with only a small concentration required to inhibit these enzymes [38]. Molecular docking further affirmed the *in vitro* inhibitory mechanisms as more identified compounds bound to the active site than the allosteric site signifying a preference for the (E) to elicit their potential pharmacological action [39]. The common interaction between Trp, Tyr, Ile, Ala, and Asp in the binding pockets of the enzymes and ligands (acarbose, vitamin E, and phytol) suggests nonpolar bonds (van der Waals force) are the major interactions occurring between the extracts and enzymes. Trp and Asp have previously been identified as common amino acids stabilising the interactions between glucosidase and various ligands, while Tyr was reported for amylase [39–41].

## 5. Conclusion

This is the first time, to the best of our knowledge, the inhibitory mechanism of *T. catappa* leaf extracts on glucosidase and amylase is being reported, making it an effective agent in managing postprandial hyperglycaemia. These extracts preferably bind to the active site of these enzymes where their various identified compounds synergistically illicit their inhibitory action. From the different GC-MS identified compounds, vitamin E was the most potent ligand that qualified as a potential drug candidate after docking studies. These plants can be leveraged upon as a natural source of not only vitamin E but other antidiabetic compounds for drug formulation. On the other hand, isolation and characterisation of these identified phytocompounds in addition to *in vivo* studies are still required to confirm these findings.

## Figures and Tables

**Table 1:** IC_50_, *V*_max_, and *K*_m_ values of *T. catappa* leaf extracts on *α*-glucosidase and *α*-amylase.

	*α*-Glucosidase			*α*-Amylase		
	IC_50_ (mg/mL)	*V*_max_ (mM/min)	*K*_m_ (mM)	IC_50_ (mg/mL)	*V *_max_ (mM/min)	*K*_m_ (mg)
TCE	3.78 ± 0.26^c^	0.13	0.19	0.24 ± 0.08^a^	0.013	2.27
TCA	3.28 ± 0.47^b^	0.10	0.77	0.75 ± 0.14^b^	0.016	2.22
Acarbose	2.23 ± 0.21^a^	—	—	0.85 ± 0.18^b^	—	—
Control	—	0.35	0.19	—	0.025	0.43

Data are represented as mean ± SD (*n* = 3). Values with different superscripts down a column are significantly different at *p* < 0.05. IC_50_: half maximal inhibitory concentration; *V*_max_: maximum velocity; *K*_m_: Michaelis constant.

**Table 2:** GC-MS identified phytochemicals present in *T. catappa* ethanolic leaf extract.

Peak no.	Compound name	Retention time (min)	Area (%)	Molecular weight (g/mol)	Formula	Classification of compound
1	2-Furancarboxaldehyde, 5-methyl-	7.227	0.05	110.11	C_6_H_6_O_2_	Carbohydrate
2	4H-Pyran-4-one, 2,3-dihydro-3,5-dihydroxy-6-methyl-	10.034	0.54	144.12	C_6_H_8_O_4_	Phenolics
3	2,5-Dimethyl-1-hepten-4-ol	10.839	0.1	142.24	C_9_H_18_O	Terpene
4	Benzofuran, 2,3-dihydro-	11.084	0.61	120.15	C_8_H_8_O	Phenolics
5	Cyclopentanol, 1-(1-methylene-2-propenyl)-	11.235	0.24	138.21	C_9_H_14_O	Terpene
6	2-Methoxy-4-vinylphenol	12.326	0.2	150.17	C_9_H_10_O_2_	Phenolics
7	7-Oxabicyclo[4.1.0]heptane, 1,5-dimethyl-	12.417	0.08	126.20	C_8_H_14_O	Phenolics
8	1-Tetradecanol	13.133	0.09	214.39	C_14_H_30_O	Fatty acid
9	*cis*-Z-*α*-Bisabolene epoxide	13.6	0.07	220.35	C_15_H_24_O	Terpenoid
10	2-(3,3-Dimethyl-but-1-ynyl)-1,1-dimethyl-3-methylene-cyclopropane	13.673	0.1	162.27	C_12_H_18_	Hydrocarbon
11	Phenol, 2,6-bis(1,1-dimethylethyl)-	14.429	0.35	206.32	C_14_H_22_O	Phenolics
12	2(4H)-Benzofuranone, 5,6,7,7a-tetrahydro-4,4,7a-trimethyl-, (R)-	14.851	0.15	180.24	C_11_H_16_O_2_	Phenolics
13	10-Heneicosene (c,t)	15.143	0.33	294.60	C_21_H_42_	Hydrocarbon
14	Ethyl-*α*-D-glucopyranoside	15.863	10.38	208.21	C_8_H_16_O_8_	Carbohydrate
15	6-Methyl-cyclodec-5-enol	16.876	0.59	168.28	C_11_H_20_O	Phenolics
16, 17	Phytol, acetate	17.09	6.92	338.60	C_22_H_42_O_2_	Terpenoid
20	9-Octadecene, 1-methoxy-, (E)-	17.88	0.25	282.50	C_19_H_38_O	Hydrocarbon
21	*n*-Hexadecanoic acid	18.072	8.95	256.43	C_16_H_32_O_2_	Fatty acid
22	Hexadecanoic acid, ethyl ester	18.172	6.83	284.50	C_18_H_36_O_2_	Fatty acid ethyl ester
23	Vitamin E	18.478	6.25	430.70	C_29_H_50_O_2_	Terpenoid
18, 19, 24	Phytol	18.985	29.54	296.50	C_20_H_40_O	Phytosterol
25	9,12-Octadecadienoic acid (Z,Z)-	19.257	2.46	280.40	C_18_H_32_O_2_	Fatty acid
26	Oleic acid	19.293	17.1	282.50	C_18_H_34_O_2_	Fatty acid
27	4-Decenoic acid, ethyl ester, (Z)-	19.425	3.79	198.30	C_12_H_22_O_2_	Fatty acid ethyl ester

**Table 3:** GC-MS identified phytochemicals present in *T. catappa* aqueous leaf extract.

Peak no.	Compound	Retention time (min)	Area (%)	Molecular weight (g/mol)	Formula	Classification of compound
1	2,3-Butanediol	6.399	2.14	90.12	C_4_H_10_O_2_	Alcohol
2	Diglycerol	8.958	3.31	166.17	C_6_H_14_O_5_	Fatty acid
3	4H-Pyran-4-one, 2,3-dihydro-3,5-dihydroxy-6-methyl-	10.105	2.03	144.12	C_6_H_8_O_4_	Phenolics
4	Benzofuran, 2,3-dihydro-	11.115	1.49	120.15	C_8_H_8_O	Phenolics
5	2-Methoxy-4-vinylphenol	12.334	0.98	150.17	C_9_H_10_O_2_	Phenolics
6	1,2,3-Benzenetriol	13.444	9.63	126.11	C_6_H_6_O_3_	Phenolics
7	1,2,4-Benzenetriol	13.58	4.65	126.11	C_6_H_6_O_3_	Phenolics
8	2-Cyclohexen-1-one, 3-(hydroxymethyl)-6-(1-methylethyl)-	14.279	0.92	168.23	C_10_H_16_O_2_	Terpenoid
9	9-Oxabicyclo[3.3.1]nonane-2,6-diol	15.295	11.02	158.19	C_8_H_14_O_3_	Phenolics
10, 25	9,9-Dimethoxybicyclo[3.3.1]nona-2,4-dione	15.448	3.36	212.24	C_11_H_16_O_4_	Phenolics
13, 15	9,10-Secocholesta-5,7,10(19)-triene-1,3-diol, 25-[(trimethylsilyl)oxy]-, (3*β*,5Z,7E)-	15.873	1.61	212.24	C_30_H_52_O_3_Si	Terpenoid
14	8-Methyl-6-nonenoic acid	16.111	1.12	170.25	C_10_H_18_O_2_	Fatty acid
11, 12, 16–19	[1,1′-Bicyclopropyl]-2-octanoic acid, 2′-hexyl-, methyl ester	16.942	5.2	322.50	C_21_H_38_O_2_	Fatty acid methyl ester
21	4-Decenoic acid, 3-methyl-, (E)-	17.322	1.39	184.27	C_11_H_20_O_2_	Fatty acid
23	Cycloheptanone imine, 2,2,7,7-tetramethyl-	17.488	2.52			Alkaloid
24	*n*-Hexadecanoic acid	18.049	6.77	256.43	C_16_H_32_O_2_	Fatty acid
26	Vitamin E	18.523	6.33	430.70	C_29_H_50_O_2_	Terpenoid
27	Jasmolin II	18.572	0.15	374.50	C_22_H_30_O_5_	Pyrethrin
20, 22, 28	Phytol	18.964	2.77	296.50	C_20_H_40_O	Phytosterol
29	9,12-Octadecadienoic acid (Z,Z)-	19.214	6.39	280.40	C_18_H_32_O_2_	Fatty acid
30	17-Octadecynoic acid	19.318	8.31	167.29	C_11_H_21_N	Fatty acid

**Table 4:** Virtual screening results of identified ligand on *α*-amylase using iGEMDOCK.

S. no	Compound	(kcal/mol)			
		TE	VdW	Hbond	Elec
1	[1,1-Bicyclopropyl]-2-octanoicacid, 2-hexyl-, methyl ester-	−71.55	−71.55	0.00	0.00
2	1,2,3-Benzenetriol	−62.38	−47.42	−14.96	0.00
3	Ethyl-*α*-D-glucopyranoside	−78.11	−55.12	−22.99	0.00
4	Hexadecanoic acid, ethyl ester	−65.40	−60.40	−5.00	0.00
5	*n*-Hexadecanoic acid	−65.71	−45.93	−16.41	−3.37
6	Oleic acid	−71.75	−51.69	−16.66	−3.41
7	Phytol acetate	−67.32	−66.64	−0.68	0.00
8	Phytol	−64.40	−53.90	−10.50	0.00
9	Vitamin E	−82.91	−76.90	−6.01	0.00
10	9,12-Octadecadienoic acid (Z,Z)-	−68.67	−59.76	−7.33	−1.61
11	9-Oxabicyclo[3.3.1]nonane-2,6-diol	−62.20	−37.56	−24.64	0.00
12	17-Octadecynoic acid	−74.92	−66.04	−9.25	0.37
13	Acarbose	−126.81	−64.99	−61.83	0.00

TE: total energy; VdW: van der Waals bond; Hbond: hydrogen bond; Elec: electrostatic bond.

**Table 5:** Virtual screening results of identified ligand on *α*-glucosidase using iGEMDOCK.

S. no	Compound	(kcal/mol)			
		TE	VdW	Hbond	Elec
1	9,12-Octadecadienoic acid (Z,Z)-	−74.89	−72.86	0.00	−2.02
2	9-Oxabicyclo[3.3.1]nonane-2,6-diol	−65.03	−46.52	−18.51	0.00
3	17-Octadecynoic acid	−71.74	−69.29	−1.90	−0.56
4	[1,1-Bicyclopropyl]-2-octanoicacid, 2-hexyl, methyl ester	−66.96	−64.46	−2.50	0.00
5	1,2,3-Benzenetriol	−70.52	−46.14	−24.38	0.00
6	Ethyl-*α*-D-glucopyranoside	−79.92	−53.16	−26.76	0.00
7	Hexadecanoic acid, ethyl ester	−69.78	−60.29	−9.49	0.00
8	*n*-Hexadecanoic acid	−81.89	−70.45	−11.44	0.00
9	Oleic acid	−76.72	−62.87	−13.84	0.00
10	Phytol acetate	−70.23	−70.23	0.00	0.00
11	Phytol	−80.87	−72.93	−7.95	0.00
12	Vitamin E	−89.64	−89.64	0.00	0.00
13	Acarbose	−115.55	−78.78	−36.77	0.00

TE: total energy; VdW: van der Waals bond; Hbond: hydrogen bond; Elec: electrostatic bond.

**Table 6:** Drug-likeness violation of selected virtual screened hit compounds.

S. no	Compound	MW	Log *P*	HA	HD	# Lipinski violations
1	Ethyl-*α*-D-glucopyranoside	208.21	−2.18	6	4	—
2	*n*-Hexadecanoic acid	256.42	4.19	2	1	—
3	Phytol	296.53	5.25	1	1	1
4	Vitamin E	430.71	6.14	2	1	1
5	Acarbose	645.6	−6.94	19	14	3
6	Lipinski rule details	≤500	≤5	≤10	≤5	

MW: molecular weight; log *P*: octanol-water partition coefficient; HA: hydrogen acceptor; HD: hydrogen donor.

**Table 7:** Molecular docking analysis showing binding affinity, inhibition constant, and interacting residues in the binding site of *α*-amylase and *α*-glucosidase.

Protein	Compound	BE (kcal/mol)	*K*_*i*_ (*μ*M)	Hb-IR	VdWb-IR	*π*b-IR
*α*-Amylase	Ethyl-*α*-D-glucopyranoside	−6.0	40.51	Arg 361, Arg 282, Asp 332, Ile 327, Gln 317, Gly 319	Leu 328, Trp 331, Thr 329, Asn 316, Arg 318, Phe 363, Ala 325	—
	Vitamin E	−8.0	1.39	—	Gln 78, Trp 74, Asp 315, Val 249, Glu 248, His 320	Val 178, Leu 180, Leu 177, His 314, Trp 73, Tyr 77, Tyr 166, Ile 250, Ala 213
	Acarbose	−8.3	0.84	Gly 321, His 320, Asp 212, Arg 210, Glu 248, Lys 215	His 216, Asp 315, Asp 251, His 314, Gln 78, Trp 73, Trp 74, Tyr 77, Leu 180, His 116, Ala 213, Ala 322, Ile 250, Tyr 166, Glu 255	Leu 177, Val 178

Glucosidase	Ethyl-*α*-D-glucopyranoside	−5.1	184.70	Gln 743, His 742	Val 740, Val 763, Val 755, Thr 768, Thr 753, Gly 765, Pro 754, Leu 756, Gln 757	Trp 804
	*n*-Hexadecanoic acid	−5.2	156.05	Val 358	Leu 195, Leu 577, Leu 574, Leu 565, Gly 605, Ala 604, Ala 582, Tyr 609, Pro 194, Thr 578, Thr 491, Phe 490, Arg 585	Leu 496, Ile 581
	Phytol	−5.5	94.11	—	Asp 282, Asp 616, Asp 404, Asp 443, Arg 600, Ile 441, Leu 405, Leu 650, Ser 676	Phe 525, Phe 649, Trp 481, Trp 376, His 674, Ala 284, Met 519,
	Vitamin E	−5.9	47.95	—	Arg 281, Arg 500, Ala 284, Ser 523, Met 519, Phe 649, Asp 616	Trp 376, Trp 481, Leu 283, Phe 525, Asp 262
	Acarbose	−7.4	3.83	Asn 524, Asp 282, Asp 404, Asp 616, Asp 518, Arg 600, Ser 676, Trp 481	—	Ala 284

BE: binding energy; *K*_*i*_: inhibition constant; Hb-IR: hydrogen bond interacting residues; VdWb-IR: van der Waals bond interacting residues; *π*b-IR: pi bond interacting residues.

## Data Availability

The data used to support the findings of this study are included in the article.
